# Joint Source and Relay Beamforming Design in Wireless Multi-Hop Sensor Networks with SWIPT

**DOI:** 10.3390/s19010182

**Published:** 2019-01-06

**Authors:** Xiaoqing Liu, Zhigang Wen, Dan Liu, Junwei Zou, Shan Li

**Affiliations:** Beijing Key Laboratory of Work Safety Intelligent Monitoring, School of Electronic Engineering, Beijing University of Posts and Telecommunications, Beijing 100876, China; xqliu0723@163.com (X.L.); dandanmessage@126.com (D.L.); buptzjw@bupt.edu.cn (J.Z.); lish01@ehualu.com (S.L.)

**Keywords:** amplify-and-forward, beam-forming, diagonalization, energy harvesting, multi-hop, power splitting, simultaneous wireless information and power transfer, time switching, wireless sensor networks

## Abstract

We consider a multiple-input multiple-output amplify-and-forward wireless multiple-hop sensor network (WMSN). The simultaneous wireless information and power transfer technology is deployed to potentially achieve an autonomous system. We investigate two practical receiver schemes, which are the power splitting (PS) and the time switching (TS). The power splitting receiver splits received signals into two streams, one for information decoding (ID) and the other for energy harvesting (EH). On the other hand, the time switching receiver only serves in ID mode or energy harvesting mode during a certain time slot. Subject to transmit power constraints and destination harvested energy constraint, we aim to obtain a joint beam-forming solution of source and relay precoders to maximize the maximum achievable rate of the WSN. In order to make the non-convex problem tractable, diagonalization-based alternating optimization algorithms are proposed. Numerical results show the convergence and good performance of the proposed algorithms under both PS and TS protocols.

## 1. Introduction

Wireless sensor networks (WSNs) are typically supplied by batteries with limited energy [1], which becomes a severe restriction on the distance and amount of the information to be transmitted. The energy harvesting technology has been focused on renewable energy resources such as solar energy, bioenergy and wind energy originally, which provides a potential long-term operation of WSNs, reducing the cost of battery charging or replacement of sensor nodes [2,3]. Whereafter, the research on energy harvesting gradually expanded to the electromagnetic field. One of the earliest pioneers of wireless charging technology and related research was the American physicist Tesla, whose experiments were carried out in 1890s [4].

In 2008, Varshney proposed a tradeoff scheme between rate versus energy in the simultaneous wireless information and power transfer (SWIPT) system [5]. Applying SWIPT technology, the communication range can be up to several kilometers and it has promising applications in several areas that can benefit from ultra-low-power sensing devices [6]. For WSNs, SWIPT can make sensor nodes harvest energy and exchange useful information via the same radio-frequency (RF) signal [7]. Due to its convenient deployment and green environmental protection characteristics, the SWIPT technology has received more and more attention [8]. There are two practical receiver schemes applied in SWIPT systems: power splitting (PS) scheme and time switching (TS) scheme [9]. The performance of SWIPT is boosted by employing efficient receiver structures both under PS and TS receiver designs for dual-hop massive MIMO relay networks [10]. Employing PS receivers at source nodes, a problem is formulated to minimize the mean-square-error (MSE) by jointly optimizing the source-relay beam-forming matrices in a MIMO two-way relay network in ref. [11]. In ref. [12], a full-duplex two-hop MIMO system is considered based on the amplify-and-forward (AF) relay protocol, with PS applied. The achievable rate region of wirelessly powered two-way communication with nonlinear energy harvested model under a fixed and mobile relay is investigated in ref. [13]. The authors proposed two schemes to derive the joint optimal design of transceiver beamformers based on the minimum MSE criterion. As for TS protocol, joint transceiver is optimized in ref. [14] for a two-hop nonregenerative relay system, where the relay node relies on harvesting the RF energy transferred from the source node using TS protocol to forward information from source to destination. In ref. [15], a practical non-linear energy harvesting model and design a resource allocation algorithm for SWIPT systems. Reference [16] reviews and discusses recent progress in laying the foundations of wireless information and power transfer networks.

To date, most studies on SWIPT technology have been concentrated on single-hop or dual-hop systems. However, wireless multi-hop sensor networks (WMSNs), a sensor node transmits sensing data through a chain of other sensor nodes to a data station, can gather sensing data more efficiently [17]. From a practical point of view, one of the main benefits of relaying is to extend the network coverage without additional infrastructure, for which multi-hop relaying plays an important role, as the extended coverage often increases with the number of hops used [18]. Thus, in the case of a long source-destination distance, multiple relay nodes may be necessary to relay signals from the source node to the destination node [19]. The optimal source and relay matrices are jointly diagonalized in a multi-hop MIMO relay system with any number of hops, which is a further generalization of two-hop systems in ref. [19]. In ref. [20], a method to cancel interference at receivers in cache-enabled wireless stochastic networks is proposed. Applying energy harvesting (EH) technology into multi-hop systems, an energy-efficient power manager is proposed in multi-hop wireless sensor networks powered by periodic energy harvesting sources [3]. While authors of [21] conducted research on the multi-hop system with energy harvesting relays, there is no integration with MIMO technology. The MIMO technology can significantly enhance the performance of SWIPT systems in terms of the information and energy transmission [21]. Thus, there is a serious lack of theoretical research on the joint manner for MIMO system and SWIPT technologies in WMSNs.

Since a linear model for the energy harvester has the benefit of being analytically easily tractable, a MIMO AF relay WMSN with SWIPT involved is considered in this paper. Based on the PS receiver and the TS receiver schemes seperately, we study the maximization of the maximum achievable rate (MAR) when the transmission power and the harvested energy are all constrained. In order to derive the joint design of beam-forming matrices, we propose a diagonalization-based alternating optimization (AO) scheme utilizing the singular value decomposition (SVD) technique. The numerical results are presented to show the MAR performance of both receiver schemes with different parameter settings.

The remainder of the paper is organized as follows. Section 2 characterizes the system including the deployment of the sensor nodes and the transmission process. In Section 3, we establish the joint source and relay beam-forming problem subject to the transmission constraints and the harvested energy constraint for both PS and TS cases, seperately. In Section 4, the diagonalization-based AO scheme is proposed to derive the solution. Numerical results and discussion are given in Section 5. Section 6 concludes the whole paper.

***Notation:*** Throughout this paper, the boldface uppercase letters are used to denote matrices and boldface lowercase letters denote vectors. ${\left(\cdot \right)}^{T}$ is the transpose operation and ${\left(\cdot \right)}^{H}$ is the conjugate transpose operation. Tr(·) is the trace of a matrix, E[·] denotes the expectation operation and |·| is the determinant operation of a matrix.

## 2. System Model

**Figure 1:**
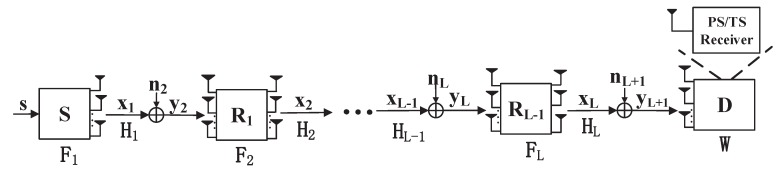
A multi-hop MIMO system with power splitting or time switching receiver.

We consider a MIMO AF relay WMSN consisting of one source node, one destination node and L−1 relay nodes, as shown in Figure 1. Without loss of generality, we assume that all the nodes are equipped with N>1 antennas. The signal, holding information, is transformed from the source node S to the destination node D, through the relay nodes $R_{1},R_{2},\ldots ,R_{L}$. Considering a worst case condition, we ignore the direct link among S, $R_{i}$ and D, i∈(1,L) as also assumed in refs. [22,23,24]. Moreover, the hardware impairments are be assumed to be ignorable since the transceivers in this system are implemented under half-duplex (HD) mode [25]. Note that D can harvest energy and decode information simultaneously through PS or TS protocol in this system. Assuming that the original complex symbol vector is $s\in C^{N\times 1}$, the signal received by the first relay $R_{1}$ is

(1) $$\begin{array}{c} x_{1}=F_{1}s \end{array}$$

where $F_{1}\in C^{N\times N}$ denotes the source beam-forming matrix. The symbol vector s satisfies $E[ss^{H}]=1$, where E[·] represents the statistical expectation arithmetic and ${\left(\cdot \right)}^{H}$ stands for the Hermitian transpose. Considering the practical limitation, we set the transmit power constraint at S as

(2) $$\begin{array}{c} \mathrm{Tr}\left(F_{1}F_{1}^{H}\right)\leq p_{1} \end{array}$$

where Tr(·) is the trace of a matrix and $p_{1}$ is the maximum transmission power at S.

The received signal at $R_{i-1}$ can be written as

(3) $$\begin{array}{c} y_{i}=H_{i-1}x_{i-1}+n_{i},i\in \left(1,L\right], \end{array}$$

where $H_{i-1}$ represents the corresponding channel matrix between the (i−1)th and the *i*th nodes and full channel state information (CSI) is assumed to be available at each node. In practice, the channel state information can be obtained during the handshaking between the transmitter and the receivers before power and data transfer start [15]. $x_{i-1}$ denotes the signal amplified and forwarded by the (i−2)th relay node. $n_{i}$ are N×1 additive white Gaussian noises (AWGNs) with normalized power superimposed before $R_{i-1}$ and D.

Then, $R_{i-1}$ multiplies $y_{i}$ by its beam-forming matrix $F_{i}$ and forward

(4) $$\begin{array}{c} x_{i}=F_{i}y_{i}=F_{i}\left(H_{i-1}x_{i-1}+n_{i}\right) \end{array}$$

to the next sensor node. Accordingly, the transmission power at $R_{i}$ should satisfy

(5) $$\begin{array}{c} \mathrm{Tr}\{F_{i}\left(\sum_{l=1}^{i-1}\left(\prod_{j=i-1}^{l}\left(H_{j}F_{j}\right)\prod_{j=l}^{i-1}\left(F_{j}^{H}H_{j}^{H}\right)+I_{N}\right)F_{i}^{H}\right\}\leq p_{i} \end{array}$$

where $p_{i}$ is the maximum transmission power at $R_{i}$. After amplified and forwarded by the L−1 relay nodes, the received signal at D is

(6) $$\begin{array}{c} {\tilde{y}}_{L+1}=H_{L}x_{L}+n_{L+1}=\cdots =\tilde{H}s+\tilde{n}+n_{L+1} \end{array}$$

where $\tilde{H}=H_{L}F_{L}\cdots H_{2}F_{2}H_{1}F_{1}=\prod_{i=L}^{1}H_{i}F_{i}$ and $\tilde{n}=H_{L}F_{L}\cdots H_{2}F_{2}n_{2}+\cdots +H_{L}F_{L}n_{L}=\sum_{l=2}^{L}\left(\prod_{i=L}^{l}H_{i}F_{i}n_{i}\right)$ denote the equivalent channel gain and noise vector, respectively.

In order to realize SWIPT, we consider two receiver schemes, the PS receiver and the TS receiver, at node D in the following sections. For both cases, we establish the optimal problem for maximizing the MAR under the transmit power constraints of the source and relay nodes and the energy-harvesting constraint of the destination node. Since the original problems are non-convex, diagonalization-based AO schemes are proposed to derive the optimized beam-forming matrices.

## 3. Joint Beamforming Design for PS Protocol

**Figure 2:**
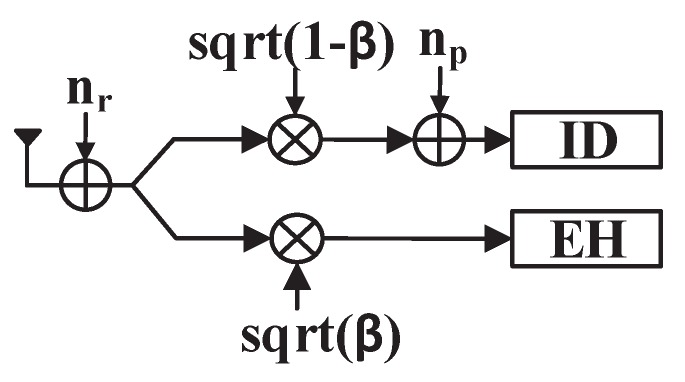
The PS receiver.

When the amplified signal is received by D, the PS receiver divides it into two streams to harvest energy and decode information simultaneously, as illustrated in Figure 2.

### 3.1. Problem Formulation

With a fixed ratio of power allocation β∈(0,1) for EH, the $\bar{\beta }=1-\beta$ portion of the received signal is assigned to the information decoding (ID) side as

(7) $$\begin{array}{c} y_{L+1}=\sqrt{\bar{\beta }}\left(\tilde{H}s+\tilde{n}+n_{L+1}+n_{r}\right)+n_{p} \end{array}$$

where $n_{r}$ and $n_{p}$ are the AWGNs with normalized power brought by the RF-band of D and caused by the power splitter, respectively. Note that the power of noise $\sqrt{\bar{\beta }}\left(n_{L+1}+n_{r}\right)+n_{p}$ is negligible compared to other terms in (7), the harvested energy should satisfy the constraint

(8) $$\begin{array}{c} \beta \mathrm{Tr}\left(\tilde{H}{\tilde{H}}^{H}+C_{n}\right)\geq e^{ps} \end{array}$$

where $C_{n}=\tilde{n}{\tilde{n}}^{H}=\sum_{l=2}^{L}\left[\prod_{i=L}^{l}\left(H_{i}F_{i}\right)\prod_{i=l}^{L}\left(F_{i}^{H}H_{i}^{H}\right)\right]$ is the covariance matrix of the equivalent noise $\tilde{n}$. The power threshold $e^{ps}$ ranges between $\left(0,e_{max}^{ps}\right)$, where $e_{max}^{ps}$ can be reached when the ratio for EH β is set to 1 [26].

The signal after ID can be calculated as

(9) $$\begin{array}{c} y_{ps}=\sqrt{\bar{\beta }}W\left(\tilde{H}s+\tilde{n}+n_{L+1}+n_{r}\right)+Wn_{p}. \end{array}$$

Assuming that the different noises are pairwise orthogonal, we can obtain the covariance matrix of the received signal as

(10) $$\begin{array}{c} R_{y}^{ps}=W\left(\bar{\beta }\tilde{H}{\tilde{H}}^{H}+Z\right)W^{H} \end{array}$$

where $Z=\bar{\beta }C_{n}+\left(2\bar{\beta }+1\right)I_{N}$. Then, the covariance matrix of the final equivalent noise can be expressed as

(11) $$\begin{array}{c} R_{n}^{ps}=WZW^{H}. \end{array}$$

According to [27], the differential entropies of the received signal and the final equivalent noise can be obtained as

(12a) $$\begin{array}{c} H_{y}^{ps}=log_{2}(|\pi eR_{y}^{ps}\left|\right) \end{array}$$

(12b) $$\begin{array}{c} H_{n}^{ps}=log_{2}(|\pi eR_{n}^{ps}\left|\right) \end{array}$$

where |·| denotes the determinant operation. Then, using (10)–(12), the mutual information between the received signal vector and the equivalent receiver noise vector is given by

(13) $$\begin{array}{cc} I(y,n) & =H_{y}^{ps}-H_{n}^{ps}\\ & =log_{2}\left(\frac{|W\left(\bar{\beta }\tilde{H}{\tilde{H}}^{H}+Z\right)W^{H}|}{|WZW^{H}|}\right)\\ & =log_{2}\left(\right|\bar{\beta }\tilde{H}{\tilde{H}}^{H}Z^{-1}+I_{N}\left|\right). \end{array}$$

From (13), the MAR, i.e., the capacity of the MIMO channel, is formulated as

(14) $$\begin{array}{c} {\mathrm{Rate}}^{ps}=log_{2}\left(\right|\bar{\beta }\tilde{H}{\tilde{H}}^{H}Z^{-1}+I_{N}\left|\right). \end{array}$$

It is significant to study the MAR under the given restricted power constraints. Then, the entire optimal beam-forming problem can be established as

(15a) $$\begin{array}{c} \max_{{\left\{F_{i}\right\}}_{i=1}^{L}}\;\;{\mathrm{Rate}}^{ps} \end{array}$$

(15b) $$\begin{array}{c} \;s.t.\;\;\mathrm{Tr}\left(F_{1}F_{1}^{H}\right)\leq p_{1} \end{array}$$

(15c) $$\begin{array}{c} \mathrm{Tr}\left\{F_{i}\left[\sum_{l=1}^{i-1}\left(\prod_{j=i-1}^{l}H_{j}F_{j}\prod_{j=l}^{i-1}F_{i}^{H}H_{j}^{H}\right)+I_{N_{r}}\right]F_{i}^{H}\right\}\leq p_{i} \end{array}$$

(15d) $$\begin{array}{c} \;\;\;\beta \mathrm{Tr}\left(\tilde{H}{\tilde{H}}^{H}+C_{n}\right)\geq e^{ps},2\leq i\leq L. \end{array}$$

The objective maximization function (15a) is non-concave and the inequality constraint (15d) is non-convex. In order to solve such an non-deterministic polynomial-time hard (NP-hard) problem, we propose a diagonalization-based AO algorithm that offers a good balance between performance and complexity.

### 3.2. Scheme Design

To determine the structure of the beam-forming matrices $F_{i}$, we first parallelize the channels $H_{i}$ using SVD method. According to [28], the decomposed form of channels is as follows

(16) $$\begin{array}{c} H_{i}=U_{i}\Phi_{i}V_{i} \end{array}$$

where ${\left\{U_{i}\right\}}_{i=1}^{L}\in C^{N\times N}$ and ${\left\{V_{i}\right\}}_{i=1}^{L}\in C^{N\times N}$ are unitary matrices. In addition, ${\left\{\Phi_{i}\right\}}_{i=1}^{L}$ are nonnegative diagonal matrices.

Accordingly, the optimal beam-forming matrices of the nodes should have the following structure

(17) $$\begin{array}{c} F_{i}=V_{i}\Lambda_{i}U_{i-1} \end{array}$$

where ${\left\{U_{i}\right\}}_{i=0}^{L-1}$ are arbitrary N×N unitary matrices and ${\left\{\Lambda_{i}\right\}}_{i=1}^{L}$ are N×N diagonal matrices with nonnegative diagonal elements which are to be determined.

Substituting (16) and (17) into problem (15), the original problem can be simplified into

(18a) $$\begin{array}{c} \max_{{\left\{\lambda_{l,k}\right\}}_{l=1}^{L}}\;\sum_{k=1}^{N}log_{2}\left[\frac{\prod_{l=1}^{L}\bar{\beta }\lambda_{l,k}^{2}\phi_{l,k}^{2}}{\bar{\beta }\sum_{l=2}^{L}\prod_{i=l}^{L}\lambda_{i,k}^{2}\phi_{i,k}^{2}+2\bar{\beta }+1}+1\right] \end{array}$$

(18b) $$\begin{array}{c} \;s.t.\;\;\sum_{k=1}^{N}\lambda_{l,k}^{2}\leq p_{1} \end{array}$$

(18c) $$\begin{array}{c} \;\;\;\;\sum_{k=1}^{N}\lambda_{l,k}^{2}\left(\sum_{l=1}^{i-1}\prod_{j=l}^{i-1}\lambda_{j,k}^{2}\phi_{j,k}^{2}+1\right)\leq p_{l},2\leq i\leq L \end{array}$$

(18d) $$\begin{array}{c} \;\;\;\;\beta \sum_{k=1}^{N}\left(\prod_{l=1}^{L}\lambda_{l,k}^{2}\phi_{l,k}^{2}+\sum_{l=2}^{L}\prod_{i=l}^{L}\lambda_{i,k}^{2}\phi_{i,k}^{2}\right)\geq e^{ps} \end{array}$$

where $\lambda_{l,k}$ and $\phi_{l,k}$ denote the (k,k)th diagonal entries of $\Lambda_{l}$ and $\Phi_{l}$, respectively.

Consequently, the original problem (15) is converted to a scalar form. To deal with the intractable matrix multiplication and addition in problem (18), we introduce the variable substitutions as follows

(19a) $$\begin{array}{c} a_{i,k}=\phi_{i,k}^{2} \end{array}$$

(19b) $$\begin{array}{c} b_{1,k}=\lambda_{1,k}^{2} \end{array}$$

(19c) $$\begin{array}{c} b_{i,k}=\lambda_{i,k}^{2}\left(a_{i-1,k}b_{i-1,k}+1\right),2\leq i\leq L. \end{array}$$

Then, we can deal with the first term of (18d) as

(20) $$\begin{array}{c} \prod_{i=1}^{L}\lambda_{i,k}^{2}\phi_{i,k}^{2}=a_{1,k}b_{1,k}\prod_{i=2}^{L}\frac{a_{i,k}b_{i,k}}{a_{i-1,k}b_{i-1,k}+1}. \end{array}$$

Moreover, the second term of (18d) is derived as

(21) $$\begin{array}{c} \sum_{l=2}^{L}\prod_{i=l}^{L}\lambda_{i,k}^{2}\phi_{i,k}^{2}=\sum_{l=2}^{L}\prod_{i=l}^{L}\frac{a_{i,k}b_{i,k}}{a_{i-1,k}b_{i-1,k}+1}\\ =\sum_{l=2}^{L}\prod_{i=l}^{L}\frac{a_{i,k}b_{i,k}}{a_{i-1,k}b_{i-1,k}+1}+a_{1,k}b_{1,k}\prod_{l=2}^{L}\frac{a_{i,k}b_{i,k}}{a_{i-1,k}b_{i-1,k}+1}-a_{1,k}b_{1,k}\prod_{l=2}^{L}\frac{a_{i,k}b_{i,k}}{a_{i-1,k}b_{i-1,k}+1}\\ =a_{L,k}b_{L,k}-a_{1,k}b_{1,k}\prod_{i=2}^{L}\frac{a_{i,k}b_{i,k}}{a_{i-1,k}b_{i-1,k}+1}. \end{array}$$

Setting $\mu =\prod_{l=1}^{L-1}\frac{a_{l,k}b_{l,k}}{a_{l,k}b_{l,k}+1}$, problem (18) can be simplified, according to (20) and (21), as

(22a) $$\begin{array}{c} \max_{{\left\{b_{l,k}\right\}}_{l=1}^{L}}\;\sum_{k=1}^{N}log_{2}\left[\frac{\bar{\beta }a_{L,k}b_{L,k}\mu }{\bar{\beta }a_{L,k}b_{L,k}\left(1-\mu \right)+2\bar{\beta }+1}+1\right] \end{array}$$

(22b) $$\begin{array}{c} \;s.t.\;\;\sum_{k=1}^{N}b_{l,k}\leq p_{l},1\leq l\leq L \end{array}$$

(22c) $$\begin{array}{c} \;\;\;\;\beta \sum_{k=1}^{N}a_{L,k}b_{L,k}\leq e^{ps},b_{l,k}\geq 0. \end{array}$$

The objective maximization function is concave with respect to $b_{l,k}$ when $b_{i,k}\left(i\neq l\right)$ are fixed. We concentrate on alternately solving each minimization problem inspired by the idea of AO. In terms of $b_{l,k}$ (with $b_{i,k}\left(i\neq l\right)$ fixed), the subproblem minimizing $-{\mathrm{Rate}}^{ps}$ can be established as

(23a) $$\begin{array}{c} \min_{{\left\{b_{l,k}\right\}}_{l=1}^{L}}\;-\sum_{k=1}^{N}log_{2}\left[\frac{\bar{\beta }a_{L,k}b_{L,k}\mu }{\bar{\beta }a_{L,k}b_{L,k}\left(1-\mu \right)+2\bar{\beta }+1}+1\right] \end{array}$$

(23b) $$\begin{array}{c} \;s.t.\;\;\sum_{k=1}^{N}b_{l,k}\leq p_{l},1\leq l\leq L \end{array}$$

(23c) $$\begin{array}{c} \;\;\;\;\beta \sum_{k=1}^{N}a_{L,k}b_{L,k}\leq e^{ps},b_{l,k}\geq 0. \end{array}$$

Note that the function and all the constraints in (23) are convex [29]. Thus, we can use CVX solver to optimize the convex problem [30].

## 4. Joint Beamforming Design for TS Protocol

**Figure 3:**
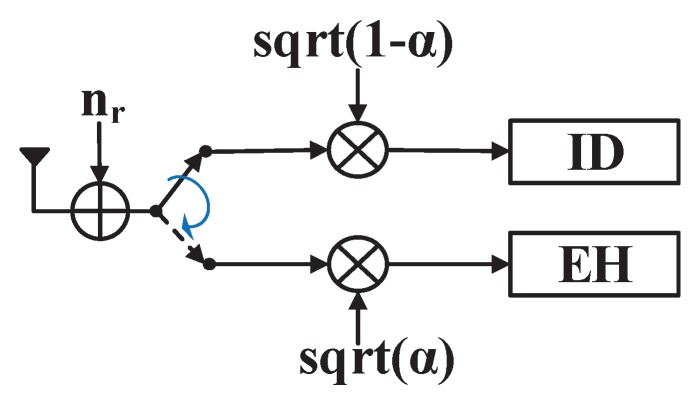
The TS receiver.

The TS protocol divides an arbitrary time block into two time slots, so that the receiver switches between ID and EH modes in different time slots according to a certain rule, as shown in Figure 3.

Setting the time switching ratio as α∈(0,1), then α portion of the transmission time is allocated for EH by the receiver. The remaining 1−α portion of time is allocated for ID.

In the ID time slot, according to (6), the covariance matrix of the received signal at D can be expressed as

(24) $$\begin{array}{c} R_{y}^{ts}=W\left(\tilde{H}{\tilde{H}}^{H}+C_{n}+2I_{N}\right)W^{H}. \end{array}$$

The covariance matrix of the final equivalent noise is

(25) $$\begin{array}{c} R_{n}^{ts}=W\left(C_{n}+2I_{N}\right)W^{H}. \end{array}$$

Following the similar steps as the PS receiver, the mutual information between the received signal and the noise can be expressed as

(26) $$\begin{array}{c} {\mathrm{Rate}}^{ts}=\left(1-\alpha \right)log_{2}\left(\right|\tilde{H}{\tilde{H}}^{H}{\left(C_{n}+2I_{N}\right)}^{-1}+I_{N}\left|\right). \end{array}$$

In the EH slot, the harvested energy at D is constrained as

(27) $$\begin{array}{c} \alpha \mathrm{Tr}\left(\tilde{H}{\tilde{H}}^{H}+C_{n}\right)\geq e^{ts} \end{array}$$

where $e^{ts}$ ranges between $\left[0,e_{max}^{ts}\right]$, where $e_{max}^{ts}$ can be reached when the ratio for EH α is set to 1 [26].

Considering the transmit power constraints at S and $R_{i}$, the entire optimal beam-forming problem under TS protocol can be established as

(28a) $$\begin{array}{c} \max_{{\left\{F_{i}\right\}}_{i=1}^{L}}\;{\mathrm{Rate}}^{ts} \end{array}$$

(28b) $$\begin{array}{c} s.t.\;\;\mathrm{Tr}\left(F_{1}F_{1}^{H}\right)\leq p_{1} \end{array}$$

(28c) $$\begin{array}{c} \mathrm{Tr}\left\{F_{i}\left[\sum_{l=1}^{i-1}\left(\prod_{j=i-1}^{l}H_{j}F_{j}\prod_{j=l}^{i-1}F_{i}^{H}H_{j}^{H}\right)+I_{N_{r}}\right]F_{i}^{H}\right\}\leq p_{i} \end{array}$$

(28d) $$\begin{array}{c} \;\;\alpha \mathrm{Tr}(\tilde{H}{\tilde{H}}^{H}+C_{n})\geq e,2\leq i\leq L. \end{array}$$

Following the same simplification steps, problem (28) can be reduced to the scalar form as

(29a) $$\begin{array}{c} \min_{{\left\{b_{l,k}\right\}}_{l=1}^{L}}\;-\sum_{k=1}^{N}log_{2}\left[\frac{a_{L,k}b_{L,k}\mu }{a_{L,k}b_{L,k}\left(1-\mu \right)+2}+1\right] \end{array}$$

(29b) $$\begin{array}{c} \;s.t.\;\;\sum_{k=1}^{N}b_{l,k}\leq p_{l},1\leq l\leq L \end{array}$$

(29c) $$\begin{array}{c} \;\;\;\;\alpha \sum_{k=1}^{N}a_{L,k}b_{L,k}\leq e,b_{l,k}\geq 0 \end{array}$$

where $\mu =\prod_{l=1}^{L-1}\frac{a_{l,k}b_{l,k}}{a_{l,k}b_{l,k}+1}$.

With *L* optimization variables ${\left\{b_{l,k}\right\}}_{l=1}^{L}$ to be dealt with, problem (29) is intractable. Notice that all the sub-problems for $b_{l,k}$ are convex with $b_{i,k}$ (i≠l) fixed. The optimal beam-forming design can be derived resorting to the MATLAB CVX toolbox. Starting with a random feasible point, the optimization of all ${\left\{b_{l,k}\right\}}_{l=1}^{L}$ is an iterative process. During the process of the iteration in the proposed diagonalization scheme, the optimized results, obtained after each iteration, serves as initial values for the next iteration. Since the results of each iteration are the optimal solutions of convex problems, the diagonalization-based AO scheme is expected to be convergent [31]. Accordingly, the computational complexity of both the proposed PS and TS scheme is calculated as $O[k_{AO}\cdot L\cdot N^{3.5}]$ in our diagonalizing design, where $k_{AO}$ denotes the number of iterations needed for the alternating optimization to converge.

## 5. Numerical Results and Disscussion

With 100 groups of randomly generated rayleigh fading channels, we conduct the numerical simulations to show the MAR performance of both the PS and TS protocol. We assume the variance of each channel is unit and $p_{1}=p_{2}=\cdots =p_{L}=p$. Assuming that the distances between adjacent nodes are equal, we study the trend of MAR along with various parameters. The carrier frequency of the system is given as f=5 GHz as set in ref. [32].

**Figure 4:**
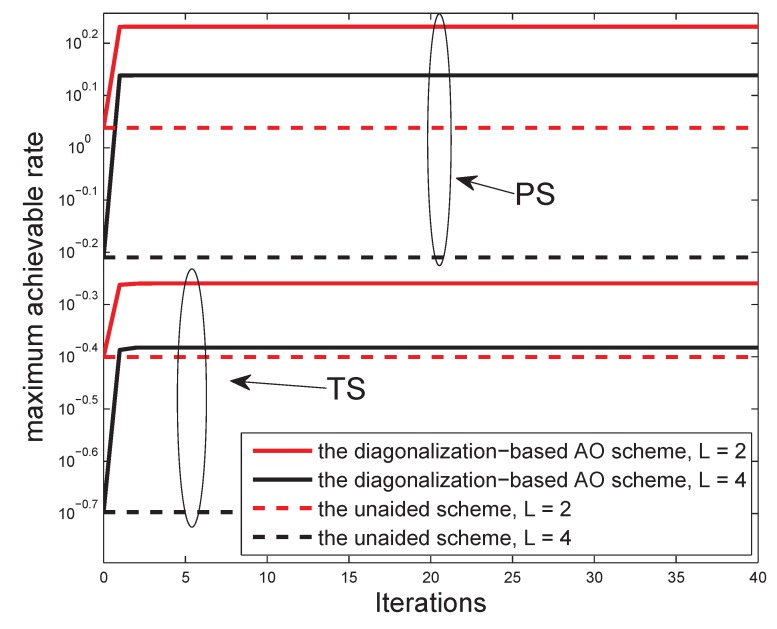
The MAR performance versus the number of iterations for N=2 and L∈{2,4}.

We can see the convergence trend of the proposed diagonalization-based AO schemes in Figure 4. The unaided scheme, obtained by a random initial point, is set as a workbench for comparison. As shown in Figure 4, the diagonalization-based AO scheme can be convergent in almost one or two iterations owing to its low-complexity scalar form, as expected. We can also see that the more number of hops, the smaller the MAR. That is, the multi-hop communication network with SWIPT sacrifices the data rate for the extension of network cover area. However, the MAR performance is apparently improved by the proposed algorithm compared to the unaided scheme in both protocols. Hence, in actual situations, it is essential to weigh the information rate against the cover area.

**Figure 5:**
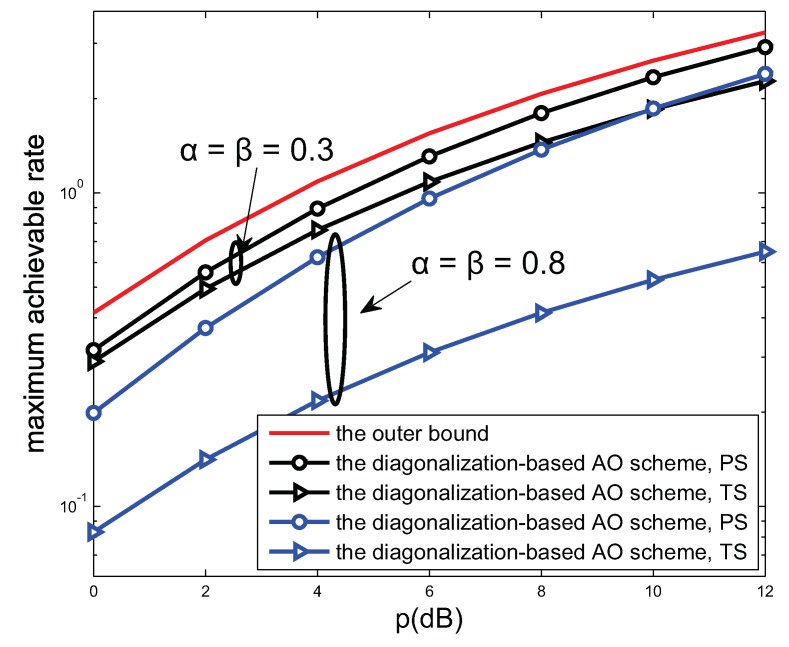
The MAR performance versus *p* of the proposed schemes for N=2, L=4 and α=β∈{0.3,0.8}.

Figure 5 presents the trend of the MAR along with the transmission power outer bound. The outer bound of the system is obtained by setting the EH ratio α=β=0, through which the system is actually transformed into a wireless information transmission (WIT) mode as investigated in ref. [11]. It can be seen from Figure 5 that the augment of value of *p* increases the MAR. Meanwhile, we set the ratio for EH in the PS and TS mode as 0.3 and 0.8 separately. When the parameters α and β increase, the MAR tend to decrease. Note that the solution obtained from AO method is not optimal but is not far from the outer bound. It can be concluded that the harvested energy and the MAR are mutually exclusive concepts. In practical scenarios, the allocation ratio should be balanced between the MAR and the harvested energy.

## 6. Conclusions

The PS receiver and the TS receiver protocols were investigated for a MIMO AF relay WMSN with SWIPT. We established a joint optimal problem under the transmission power constraints and the harvested energy constraint. With the desire to study the maximum achievable rate, the diagonalization-based alternating optimization schemes were proposed to drive the joint design of beam-forming matrices. The numerical results show the convergence and good performance of the proposed schemes. We also provided practical insights into the dependence of the maximum achievable rate on the number of hops, the transmission power outer bound and the EH ratio.

## Abbreviations

The following abbreviations are used in this manuscript:

WSN	Wireless sensor network
SWIPT	Simultaneous wireless information and power transfer
RF	Radio-frequency
PS	Power splitting
TS	Time switching
MIMO	Multiple-input multiple-output
MSE	Mean-square-error
AF	Amplify-and-forward
WMSN	Wireless multi-hop sensor network
EH	Energy harvesting
MAR	Maximum achievable rate
AO	Alternating optimization
SVD	Singular value decomposition
CSI	Channel state information
AWGN	Additive white Gaussian noise
ID	Information decoding
NP-hard	Non-deterministic polynomial-time hard
WIT	Wireless information transmission
